# Next-Generation Ultrasol Curcumin Boosts Muscle Endurance and Reduces Muscle Damage in Treadmill-Exhausted Rats

**DOI:** 10.3390/antiox10111692

**Published:** 2021-10-26

**Authors:** Emre Sahin, Cemal Orhan, Fusun Erten, Besir Er, Manutosh Acharya, Abhijeet A. Morde, Muralidhara Padigaru, Kazim Sahin

**Affiliations:** 1 Department of Animal Nutrition, Faculty of Veterinary Medicine, Bingol University, Bingol 12000, Turkey; esahin@bingol.edu.tr; 2 Department of Animal Nutrition, Faculty of Veterinary Medicine, Firat University, Elazig 23119, Turkey; corhan@firat.edu.tr; 3 Department of Veterinary Science, Pertek Sakine Genc Vocational School, Munzur University, Tunceli 62500, Turkey; fusunerten@munzur.edu.tr; 4 Department of Biology, Faculty of Science, Firat University, Elazig 23119, Turkey; 181110203@firat.edu.tr; 5 Research and Development, OmniActive Health Technologies, Mumbai 400001, India; m.acharya@omniactives.com (M.A.); a.morde@omniactives.com (A.A.M.); m.padigaru@omniactives.com (M.P.)

**Keywords:** curcumin, exhaustion, inflammation, antioxidant, exercise

## Abstract

Curcumin positively affects performance during exercise and subsequent recovery. However, curcumin has limited bioavailability unless consumed in larger doses. In the current study, we examined the impact of a new formulation of curcumin, Next-Generation Ultrasol Curcumin (NGUC), which is relatively more bioavailable than natural curcumin on exhaustion time, grip strength, muscle damage parameters, and serum and muscle proteins. A total of 28 rats were randomly grouped as control (C, non-supplemented), exercise (E, non-supplemented), E+NGUC100 (supplemented with 100 mg/kg BW NGUC), and E+NGUC200 (supplemented with 200 mg/kg NGUC). Grip strength and exhaustion time were increased with NGUC supplementation (*p* < 0.0001). Creatine kinase (CK), lactate dehydrogenase (LDH), lactic acid (LA), myoglobin, malondialdehyde (MDA) concentrations were reduced in serum, and muscle tissue in NGUC supplemented groups (*p* < 0.05). In contrast, NGUC supplementation elevated the antioxidant enzyme levels compared to the non-supplemented exercise group (*p* < 0.01). Additionally, inflammatory cytokines were inhibited with NGUC administration (*p* < 0.05). NGUC decreased PGC-1α, p-4E-BP1, p-mTOR, MAFbx, and MuRF1 proteins in muscle tissue (*p* < 0.05). These results indicate that NGUC boosts exercise performance while reducing muscle damage by targeting antioxidant, anti-inflammatory, and muscle mass regulatory pathways.

## 1. Introduction

The beneficial effects of regular physical activity on improving insulin sensitivity, limiting metabolic syndrome, reducing blood pressure, and improving muscle metabolic activity and antioxidant function have been previously established [1,2,3,4]. However, exercise-induced fatigue and muscle-soreness, if ignored, may negatively affect health and wellbeing. The oxygen utilization rate in the mitochondria increases during exercise, and it induces the release of free radicals and reactive oxygen species (ROS) in the cell. Chronic, exhaustive types of exercise routines may trigger the excessive production of ROS, inducing inflammatory reactions [5], and increasing proinflammatory cytokines [6], leading to muscle damage and fatigue [7]. Antioxidant supplementation, notable polyphenols, offers opportunities to attenuate muscle damage, fatigue, and inflammatory activity [6,8] and limit muscle atrophy [9].

Curcumin, a natural polyphenol obtained from turmeric rhizomes (*Curcuma longa*), has antioxidant, anti-inflammatory, anticancer, and immunomodulatory effects [10,11,12]. In a recent randomized clinical trial, it has been reported that exercise-induced oxidative stress, inflammatory activity, muscle soreness, and muscle damage could be reduced by 28 days of curcumin supplementation (1500 mg/day) in humans [8]. Muscle recovery activity of curcumin probably depends on the regulation of nuclear factor erythroid 2–related factor 2 (Nrf2) and nuclear factor kappa B (NF-κB) signaling [13] and the suppressing of the activity of muscle atrophy F-box (MAFbx) and muscle RING-finger protein-1 (Murf1) proteins that mediate muscle wasting [14,15].

Despite the favorable effects of curcumin on muscle function and exercise capacity, the efficiency of curcumin is still limited due to its insufficient water solubility and inadequate bioavailability [16]. Orally ingested curcumin is poorly absorbed from the intestines and mainly excreted with feces [16]. Additionally, curcumin degrades in physiological pH ranges (pH > 7), particularly in the intestinal environment [16,17,18]. Previously, to enhance curcumin bioavailability, many curcumin formulations have been invented, such as piperine (as adjuvant), liposomes, nanoparticles, and phospholipid complexes [19]. A recent study found that the bioavailability of Next-Generation Ultrasol Curcumin (NGUC) is 64.7 fold higher than natural 95% turmeric extracts in rats [12]. The superior bioavailability of NGUC vs. standard 95% turmeric extract has also been confirmed in a clinical study (manuscript in preparation). NGUC has been formulated to improve the water solubility of curcumin and facilitate its intestinal absorption by protecting it from degradation at intestinal alkaline pH conditions [12]. The current study is conducted to investigate the impacts of NGUC on muscle activity and endurance capacity. Here we demonstrated the impact of NGUC on muscle antioxidant capacity, anti-inflammatory action, muscle metabolism, strength, and endurance capacity in the treadmill-exhausted rats.

## 2. Materials and Methods

### 2.1. Animals and Design

A total of 28 Wistar Albino male rats (8 weeks, 180 ± 20 g) were kept in a controlled environment in polypropylene cages (12 light:12 dark; 22 °C). Standard rat diet and water were provided *ad libitum*. The animal study was performed according to the Care and Use of Laboratory Animals and authorized by the Animal Experiments Local Ethics Committee of the Bingöl University (Date: 13 November 2020, meeting number: 2020/04, decision number: 04/03) following the Directive 2010/63/EU.

After the adaptation period (1 week), rats were randomly distributed into four treatment groups (*n* = 7). (i) Sedentary control (C): rats were orally administered physiological saline (a volume of 1 mL per rat) without exercise and remained sedentary throughout the experiment period; (ii) Exercise (E): rats were orally administered physiological saline (a volume of 1 mL per rat) and were exercised using a rodent treadmill; (iii) E+NGUC100: rats were orally administered 100 mg/kg of NGUC (equal to 20 mg/kg of curcuminoids) and were exercised using a rodent treadmill; (iv) E+NGUC200: Rats were orally administered 200 mg/kg of NGUC (equal to 40 mg/kg of curcuminoids) and were exercised using a rodent treadmill. The duration of the study was 42 days.

NGUC was provided as a formulation of 95% curcuminoid extract by OmniActive Health Technologies (Mumbai, India), as detailed in Yabas et al. [12].

The running endurance time and distance were measured by MAY-TME (Commat Limited., Ankara, Turkey) animal treadmill. For acclimation to the actual test, the rats were pre-trained on the treadmill at 12 m/min speed on 0° inclines. The exhaustive exercise was performed on the last day of the study period. Firstly, the rats were run at the speed of 12 m/min on 0° inclines, followed by an increase of 3 m/min after that every 20 min for 60 min. Next, every 20 min, the incline of the running platform was increased by 5°. The shock grid was programmed to deliver 0.2 mA of electricity to stimulate the animals without physically injuring them. The rats were defined as exhausted when incapable of running after 10 s of electric stimulation, and exhaustion times were recorded.

The combined forelimb and hindlimb grip strength of rats was measured on the 41st day of the study period. The peak force was determined by using a force measurement system that has an electronic digital force gauge. Each rat was slightly held from the tail until it released the pull bar. After five consecutive tests, peak values were recorded.

### 2.2. Sampling

All rats were decapitated under anesthesia at the end of the study, immediately after exhaustive exercise. Next, blood and gastrocnemius muscle were collected, and blood samples were centrifuged. The gastrocnemius muscle was removed and placed on ice. The samples were kept at −80 °C until analysis. Muscle samples were homogenized in 10 volumes of cold Tris 10 mM for biochemical assays. To obtain the low-speed supernatant fraction, the homogenates were centrifuged (4000 rpm, 4 °C, 10 min).

### 2.3. Biochemical Analysis

An automated biochemical device (Samsung Electronics Co., Suwon, Korea) was used to determine the serum glucose, triglyceride (TG), total cholesterol (TC), blood urea nitrogen (BUN), alanine aminotransferase (ALT), and aspartate aminotransferase (AST) levels with rat-specific kits. Serum Creatine kinase (CK, MyBioSource, Cat No. MBS267514, San Diego, CA, USA), lactate dehydrogenase (LDH, MyBioSource, Cat No. MBS269777, San Diego, CA, USA), lactic acid (LA, MyBioSource, Cat No. MBS755975, San Diego, CA, USA), and myoglobin (MyBioSource, Cat No. MBS564122, San Diego, CA, USA) concentrations were determined through ELISA (Bio-Tek Instruments Inc., Winooski, VT, USA) according to the manufacturer’s instructions.

The serum and muscle malondialdehyde (MDA) levels were determined by High-Performance Liquid Chromatography (HPLC, Shimadzu, Tokyo, Japan) combined with an ultraviolet-visible (UV-VIS, SPD-10 AVP, Shimadzu, Tokyo, Japan) detector and C18 (Octadecyl-silica, −3, 5 μm, 4.6 mm × 250 mm) column as described previously [20]. The activities of antioxidant enzymes [superoxide dismutase (SOD), catalase (CAT), and glutathione peroxidase (GSH-Px)] in serum and muscle tissues were determined by assay kits (Cayman Chemical, Ann Arbor, MI, USA), according to the manufacturer’s instructions.

### 2.4. Protein Analyses

The level of interleukin-1β (IL-1β), IL-6, tumor necrosis factor α (TNF-α), peroxisome proliferator-activated receptor-gamma coactivator 1 alpha (PGC-1α), phosphorylated 4E-binding protein 1 (p-4E-BP1), phosphorylated mammalian target of rapamycin (p-mTOR), P70 ribosomal protein S6 kinase 1 (p70S6K1), muscle atrophy F-box (MAFbx), and muscle RING-finger protein-1 (Murf1) proteins in muscle tissue were measured by western blotting. The pooled muscle tissues were homogenated with lysis buffer. Next, the protein concentration of homogenates was quantitated by a Nanodrop spectrophotometer (Maestrogen Inc., Taiwan). An equal amount of protein loaded to sample well for sodium dodecyl sulfate-polyacrylamide gel electrophoresis (SDS-PAGE). After the electrophoresis, proteins were transferred into nitrocellulose membranes (0.45 µm); unspecific proteins were blocked by 5% bovine serum albumin. The nitrocellulose membranes were incubated with primary antibodies (IL-1β, IL-6, TNF-α, PGC-1α, p-4E-BP1, p-mTOR, p70S6K1, MAFbx, MuRF-1, Abcam, UK) overnight at 4 °C. β-actin protein was used to control protein loading. Specific binding between primary and secondary antibodies was visualized with diaminobenzidine (DAB) substrate. The protein bands were analyzed densitometrically with the Image J software (National Institute of Health, Bethesda, MD, USA). Blots were performed at least three times.

### 2.5. Statistical Analysis

The IBM SPSS software (Version 22.0, IBM Corp., Armonk, NY, USA) was used to analyze the data. Shapiro–Wilk and Levene tests were used, respectively, to check the normality of data and the homogeneity of the variances. One-way analysis of variance (ANOVA) and Tukey post-hoc test was performed to determine the changes between the groups. Means and standard error of the mean (SEM) are used to present the data.

## 3. Results

**Figure 1:**
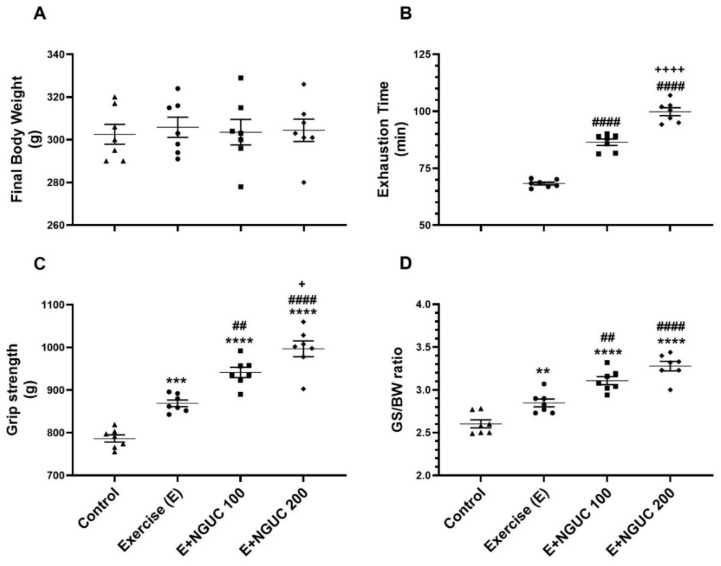
Effects of next-generation ultrasol curcumin (NGUC) on body weight (**A**), exhaustion time (**B**), grip strength (**C**) and grip strength:body weight ratio (GS:BW) (**D**) in exercised rats. The data represent the mean and standard error of the mean. Small symbols (▲,●,■,♦) shows individual values. Different symbols (*, ^#^, and ^+^ indicates difference compared to the Control, Exercise, and E+NGUC100 groups, respectively) above the groups indicate statistical differences (ANOVA and Tukey’s post-hoc test; ** *p* < 0.01, *** *p* < 0.001, **** *p* < 0.0001; ^##^ *p* < 0.01, ^####^ *p* < 0.0001; ^+^ *p* < 0.05, ^++++^ *p* < 0.0001).

Serum biochemical parameters were not affected either statistically or clinically by exercise or any dose of NGUC supplementation in rats (Table 1, *p* > 0.05). Similarly, the body weights (BW) of rats did not change with NGUC supplementation (Figure 1A, *p* > 0.05).

Exhaustion time significantly increased in the NGUC supplemented groups, particularly at the 200 mg/kg dose, compared to the exercise group (Figure 1B, *p* < 0.0001). NGUC supplementation at a dose of 100 mg/kg increased grip strength compared to the control and exercise groups (Figure 1C, *p* < 0.0001). Additionally, the E+NGUC200 group had higher grip strength than the E+NGUC100 group (*p* < 0.05). In parallel, it was observed that grip strength to BW ratio increased in curcumin supplemented groups compared to the control group (Figure 1D, *p* < 0.0001).

**Figure 2:**
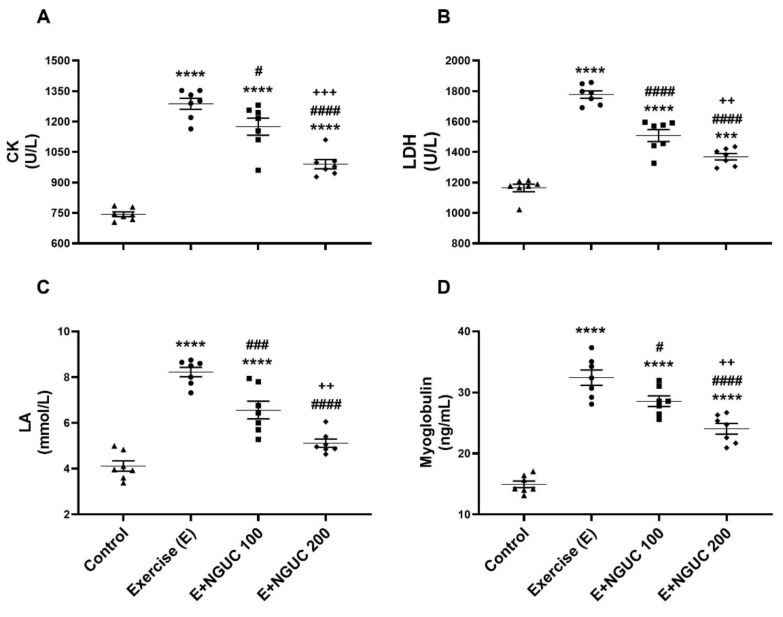
Effects of next-generation ultrasol curcumin (NGUC) on serum creatine kinase (CK) (**A**), lactate dehydrogenase (LDH) (**B**), lactic acid (LA) (**C**) and myoglobin (**D**) levels in the exercised rats. The data represent the mean and standard error of the mean. Small symbols (▲,●,■,♦) shows individual values.Different symbols (*, ^#^, and ^+^ indicates difference compared to the Control, Exercise, and E+NGUC100 groups, respectively) above the groups indicate statistical differences (ANOVA and Tukey’s post-hoc test; *** *p* < 0.001, **** *p* < 0.0001; ^#^ *p* < 0.05, ^###^ *p* < 0.001, ^####^ *p* < 0.0001; ^++^ *p* < 0.01, ^+++^ *p* < 0.001).

Blood CK (Figure 2A), LDH (Figure 2B), LA (Figure 2C), and myoglobin (Figure 2D) values raised significantly in exhausted rats as compared to the control group (*p* < 0.0001, for all). When compared to the exercise group, blood CK, LDH, LA, and myoglobin values decreased in the E+NGUC200 group (*p* < 0.0001, for all), but the E+NGUC200 group still had higher CK (*p* < 0.0001), LDH (*p* < 0.001), and myoglobin (*p* < 0.0001) values compared to the control group. Moreover, we detected that the E+NGUC200 group effectively reduced blood CK, LDH, LA, and myoglobin compared to the E+NGUC100 group (*p* < 0.01).

**Figure 3:**
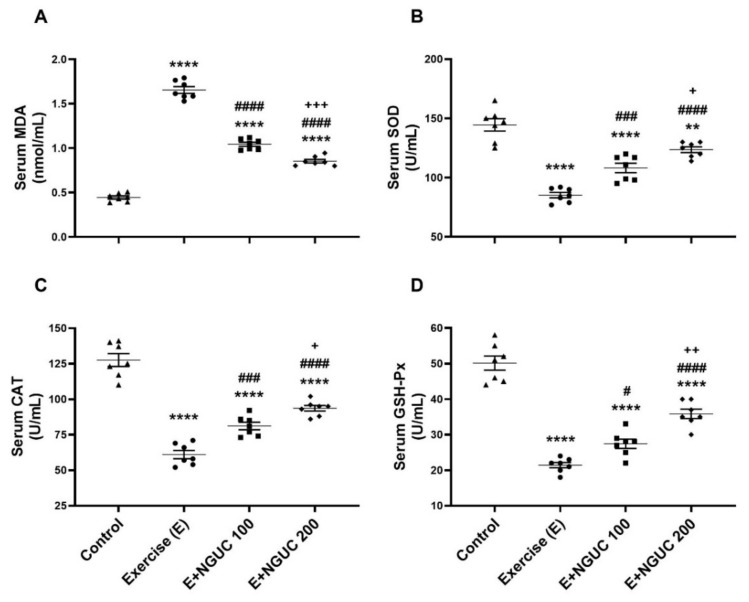
Effects of next-generation ultrasol curcumin (NGUC) on serum MDA (**A**), SOD (**B**), CAT (**C**), and GSH-Px (**D**) levels in the exercised rats. The data represent the mean and standard error of the mean. Small symbols (▲,●,■,♦) shows individual values. Different symbols (*, ^#^, and ^+^ indicates difference compared to the Control, Exercise, and E+NGUC100 groups, respectively) above the groups indicate statistical differences (ANOVA and Tukey’s post-hoc test; ** *p* < 0.01, **** *p* < 0.0001; ^#^ *p* < 0.05, ^###^ *p* < 0.001, ^####^ *p* < 0.0001; ^+^ *p* < 0.05, ^++^ *p* < 0.01, ^+++^ *p* < 0.001).

**Figure 4:**
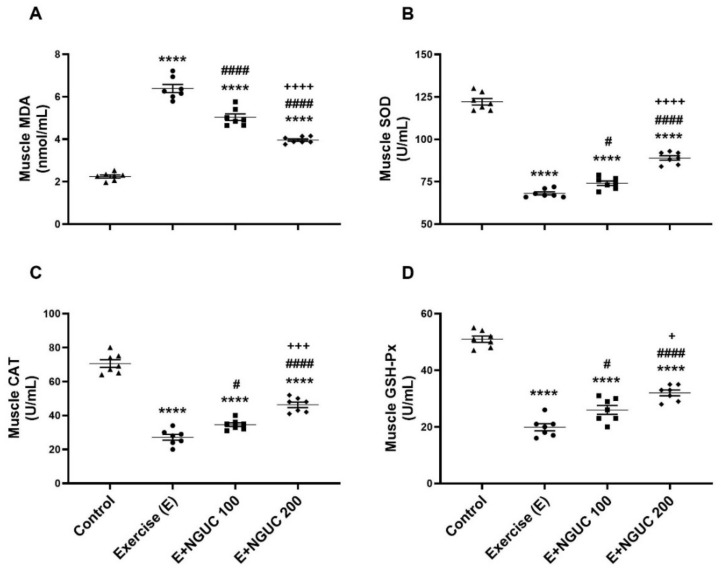
Effects of next-generation ultrasol curcumin (NGUC) on muscle MDA (**A**), SOD (**B**), CAT (**C**), and GSH-Px (**D**) levels in the exercised rats. The data represent the mean and standard error of the mean. Small symbols (▲,●,■,♦) shows individual values. Different symbols (*, ^#^, and ^+^ indicates difference compared to the Control, Exercise, and E+NGUC100 groups, respectively) above the groups indicate statistical differences (ANOVA and Tukey’s post-hoc test; **** *p* < 0.0001; ^#^ *p* < 0.05, ^####^ *p* < 0.0001; ^+^ *p* < 0.05, ^+++^ *p* < 0.001, ^++++^ *p* < 0.0001).

We detected that the MDA concentration, an oxidative stress marker, notably increased in serum and muscle in the exercise group compared to other groups (Figure 3A or Figure 4A; *p* < 0.0001, for all). A dose of 200 mg/kg NGUC supplementation effectively prevented the increase of MDA level in both serum and muscle tissue by comparison to the exercise and the E+NGUC100 groups (*p* < 0.0001, for all). Unlike the MDA levels, SOD, CAT, and GSH-Px activities decreased in both serum and muscle tissue after exhausting exercise compared to the control group (*p* < 0.0001, for all). NGUC supplementations markedly accelerated the SOD, CAT, and GSH-Px activities in the serum and the muscle tissue (Figure 3B–D or Figure 4B–D; *p* < 0.05). Particularly, when compared to the E+NGUC100 group, the E+NGUC200 group had higher SOD (*p* < 0.05 for serum and *p* < 0.0001 for muscle), CAT (*p* < 0.05 for serum and *p* < 0.001 for muscle), and GSH-Px (*p* < 0.01 for serum and *p* < 0.05 for muscle) levels.

**Figure 5:**
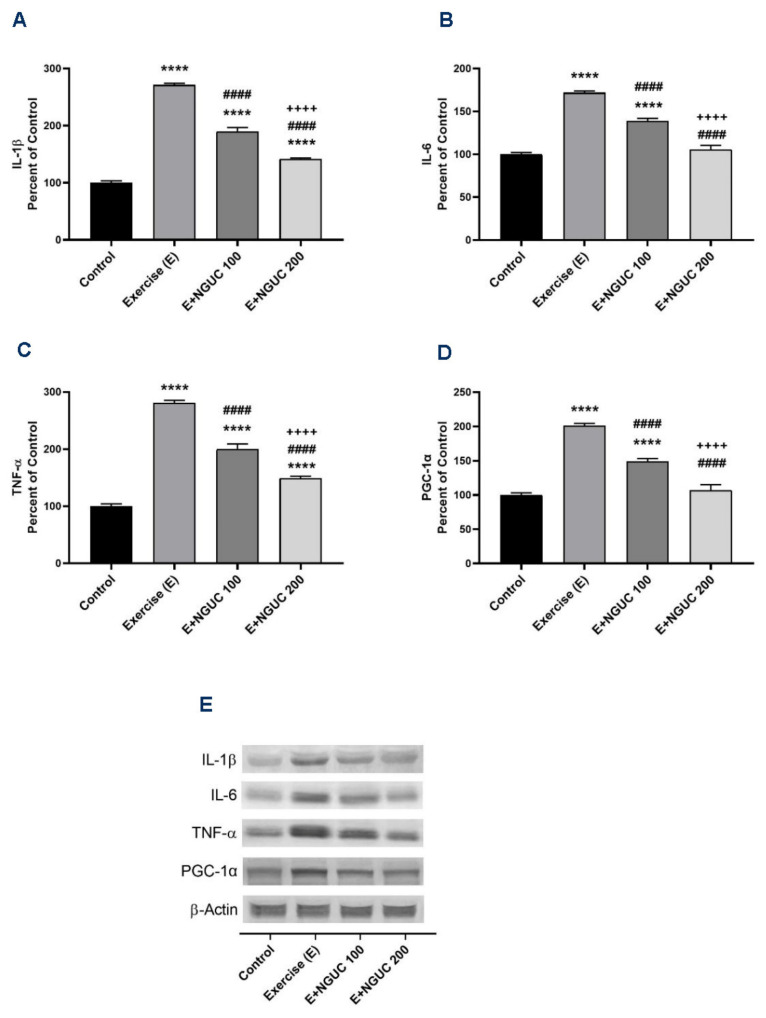
Effects of next-generation ultrasol curcumin (NGUC) on muscle IL-1β (**A**), IL-6 (**B**), TNF-α (**C**), and PGC-1α (**D**) levels in rats. Blots were repeated at least three times (*n*  =  3), and a representative blot is shown (**E**). The blots were cropped and full-length blots are presented in Supplementary Figure S1. The error lines point out the standard error of the mean. Different symbols (*, ^#^, and ^+^ indicates difference compared to the Control, Exercise, and E+NGUC100 groups, respectively) above the bars indicate statistical differences among the groups (ANOVA and Tukey’s *post-hoc test*; **** *p* < 0.0001; ^####^ *p* < 0.0001; ^++++^ *p* < 0.0001).

We found that IL-1β, IL-6, and TNF-α were significantly stimulated in muscle after exhausting exercise (Figure 5A–C), and NGUC supplementation partially reversed the inflammatory status. Compared to the control group, the muscle IL-6 level did not differ in the E+NGUC200 group (*p* > 0.05). Additionally, a significant increase was determined in exercised rats in the levels of PGC-1α, which regulates muscle adaptation metabolism (Figure 5D). The level of muscle PGC-1α gradually decreased by NGUC supplementation in a dose-dependent manner. There was no statistical difference detected between the control and E+NGUC200 group in PGC-1α levels (*p* > 0.05).

**Figure 6:**
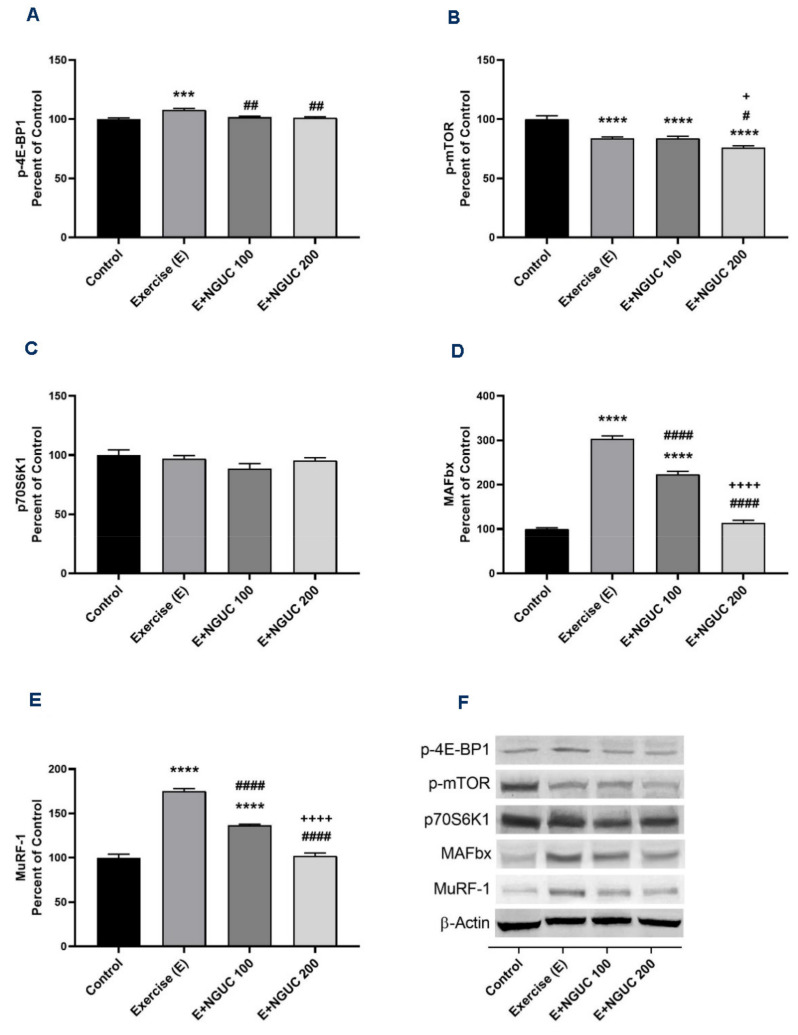
Effects of next-generation ultrasol curcumin (NGUC) on p-4E-BP1 (**A**), p-mTOR (**B**), p70S6K1 (**C**), MAFbx (**D**), and MuRF-1 (**E**) levels in rats. Blots were repeated at least three times (*n*  =  3), and a representative blot was shown (**F**). The blots were cropped and full-length blots are presented in Supplementary Figure S2. The error lines point out the standard error of the mean. Different symbols (*, ^#^, and ^+^ indicates difference compared to the Control, Exercise, and E+NGUC100 groups, respectively) above the bars indicate statistical differences among the groups (ANOVA and Tukey’s post-hoc test; *** *p* < 0.001, **** *p* < 0.0001; ^#^ *p* < 0.05, ^##^ *p* < 0.01, ^####^ *p* < 0.0001; ^+^ *p* < 0.05, ^++++^ *p* < 0.0001).

Exhaustive exercise stimulated the p-4E-BP1, MAFbx, and MuRF-1 protein levels in muscle in the exercise groups compared to the control group (Figure 6A,D,E; *p* < 0.001 for all). Compared to the exercise and the E+NGUC100 groups, the expression level of the p-mTOR protein was found lower in the E+NGUC200 group (*p* < 0.05). However, no statistical difference was detected in muscle p70S6K1 levels between groups (Figure 5C, *p* > 0.05). A dose of 200 mg/kg NGUC markedly downregulated the MAFbx and MuRF-1 protein levels by comparison to the dose of 100 mg/kg NGUC (*p* < 0.0001, for all). In addition, MAFbx and MuRF-1 protein levels did not differ between the E+NGUC200 and the control group (*p* > 0.05).

## 4. Discussion

In the current study, we used a novel curcumin formulation NGUC which is more bioavailable than natural curcumin. Serum biochemical parameters indicated that both 100 mg/kg and 200 mg/kg doses of NGUC were biologically safe, as reported in our previous study [12]. We found that NGUC increased endurance capacity and grip strength while decreasing fatigue and muscle damage, associated with oxidative stress and inflammatory status.

It has been previously reported that acute, exhaustive, and unconscious exercises lead to fatigue, muscle soreness, and muscle damage [6,7,8]. Antioxidant supplementations like curcumin could reverse the adverse effects of exercise-induced muscle damage and muscle soreness. Therefore, like our results, both human and animal studies demonstrated that different curcumin supplementations improved exhaustion time and muscle strength. [8,13,21]. The elevated CK, LDH, and myoglobin levels in serum and muscle tissue indicate muscle damage [21,22]. In addition, LA produced in muscle after exercise could cause fatigue and reduces muscle performance [23]. The reducing effect of the curcumin on the CK, LDH, myoglobin, and LA levels [13,21,22,23] may explain the boosted grip strength and extended exhaustion time in NGUC supplemented exercise rats.

The presence of the methylene group of the heptadiene-dione and/or the hydroxyl group of curcuminoids confer antioxidant and radical scavenger activity of curcumin compounds [10]. Additionally, curcumin directly targets proinflammatory cytokines (IL-1β, IL-6, and TNF-α) and thus might prevent muscle damage that adversely affects physical performance after intense exercise [24]. In this context, we demonstrated that the NGUC supplementation, particularly a 200 mg/kg dose, in both serum and muscle tissue, reduced MDA levels whereas increased antioxidant enzymes. In addition, the levels of IL-1β, IL-6, and TNF-α proteins in muscle effectively reduced in the E+NGUC200 group owing to the anti-inflammatory activity of curcumin on muscle [13,25].

PGC-1α is one of the main regulatory factors of mitochondrial biogenesis and muscle adaptation in exercise [26]. Acute exercise stimulates the PGC-1α in rodents and humans, and it helps to modulate muscle autophagy [27]. Although the previous studies reported that curcumin supplementation increased the PGC-1α protein levels or deacetylation in muscle tissue [13,28,29], we found that NGUC supplementation suppresses the muscle PGC-1α levels after one-time exhaustive exercise. Kang, et al. [30] reported that attenuating the ROS releasing with allopurinol, an antioxidant, decreased the muscle PGC-1α levels following acute exercise. This result indicated that PGC-1α signaling in skeletal muscle is sensitive to ROS activity [30]. Similarly, Khani et al. [31] showed that thyme extract supplementation reduces the muscle PGC-1α protein in exercised rats [31]. PGC-1α may initiate the production of CuZn-SOD, Mn-SOD, and GSH-Px enzymes to prevent ROS-induced muscle damage [32,33]. NGUC has a higher bioavailability and antioxidant capacity than 95% turmeric extract [12]. Therefore, muscle PGC-1α levels may have favorably decreased in the E+NGUC200 group after improving antioxidant enzyme levels.

The Akt-mTOR pathway mediates phosphorylation of 4E-BP1 and 70S6K1 proteins related to each other to maintain muscle force and protein synthesis [34]. Previous studies indicated that different type of exercise inhibits [35,36], activates [37] or did not affect the mTOR activation [38]. Many polyphenols can inhibit the mTOR and related signaling pathways [39]. In the current study, exercise inhibited phosphorylation of mTOR, and NGUC supplementation at 200 mg/kg dose further reduced its phosphorylation ratio. However, p-4E-BP1 protein level increased in the exercise group and decreased after NGUC supplementation, whereas p-70S6K1 protein levels were not affected by either exercise or NGUC supplementation. Curcumin has inhibitory effects on mTOR activation and phosphorylation of p-4E-BP1 and p-70S6K1 proteins in different cancer cell lines [40]. Additionally, Sahin et al. [41] reported that difluorinated curcumin suppressed the mTOR and its effector pathways, including p-4E-BP1 and p-70S6K1, in cisplatin-induced nephrotoxicity [41]. In addition, mTOR regulates mitochondrial biogenesis and oxidation via mediating PGC-1α [42]; thus, the mTOR/PGC-1α pathway may regulate muscle biogenesis and hypertrophy. However, the PGC-1α expression levels could be altered independently by mTOR activity in resistance-exercised rats [43]. We demonstrated that muscle PGC-1α protein level gradually decreased with reduced mTOR phosphorylation. Therefore, NGUC might have activated the mTOR-dependent PGC-1α regulation in rats.

After exhaustive exercise, the elevated inflammation and oxidative stress in our study might have triggered the production of MAFbx and MuRF1, ubiquitin E3 ligases, in muscle [44,45]. In type 1 diabetic mice, curcumin supplementation can reverse muscle atrophy by downregulating the atrogin-1/MAFbx and MuRF1 [14]. Additionally, consistent with our results, He, et al. [46] declared that curcumin alleviates muscle wasting through the inhibition of NF-κB that regulates the inflammatory reactions [46]. In addition, NGUC supplementation a dose of 200 mg/kg more effectively inhibited both MAFbx and MuRF1 protein levels, and it suggested that curcumin affects skeletal muscle in a dose-dependent manner.

## 5. Conclusions

In conclusion, our data demonstrated that NGUC boosted endurance and muscle strength and reduced muscle damage by elevating the antioxidant capacity and anti-inflammatory function by targeting specific molecular pathways. We found that curcumin regulates the mTOR-dependent activity of PGC-1α and 4E-BP1 proteins and inhibits MAFbx and MuRF1. These results suggested that NGUC contributes to improving exercise performance in rats. Nevertheless, further human clinical studies are required to validate the beneficial effect of NGUC on muscle performance and protection from muscle damage.

## Figures and Tables

**Table 1:** Effects of next-generation ultrasol curcumin (NGUC) on serum biochemical parameters in rats.

	Groups				*p*
	Control	Exercise (E)	E+NGUC100	E+NGUC200	
Glucose, mg/dL	109.86 ± 2.69	106.14 ± 2.69	105.14 ± 1.58	108.14 ± 3.33	0.603
TG, mg/dL	114.43 ± 3.71	113.86 ± 2.99	112.29 ± 3.80	110.86 ± 4.48	0.908
TC, mg/dL	133.71 ± 3.90	135.57 ± 2.95	132.29 ± 3.96	130.00 ± 3.41	0.732
BUN, g/dL	20.49 ± 0.66	20.54 ± 0.53	20.26 ± 0.32	20.13 ± 0.43	0.928
ALT, U/L	83.57 ± 2.57	87.86 ± 3.89	86.86 ± 3.38	83.57 ± 1.60	0.650
AST, U/L	105.43 ± 1.76	107.86 ± 4.73	106.57 ± 2.76	103.29 ± 3.96	0.819

TG: Triglyceride, TC: Total cholesterol; BUN: Blood urea nitrogen; ALT: Alanine aminotransferase; AST: Aspartate aminotransferase. *p* > 0.05; ANOVA and Tukey’s post-hoc test. Mean values of items are demonstrated with ± standard error of the mean.

## Data Availability

The original contributions presented in the study are included in the article/Supplementary Materials. Further inquiries can be directed to the corresponding author.

## Supplementary Materials

The following are available online at https://www.mdpi.com/article/10.3390/antiox10111692/s1, Figure S1: Full immunoblots related to Figure 5 muscle tissue, Figure S2: Full immunoblots related to Figure 6 muscle tissue.
