# Supplementary material for: Next-Generation Ultrasol Curcumin Boosts Muscle Endurance and Reduces Muscle Damage in Treadmill-Exhausted Rats
Source: Antioxidants (Basel). 2021 Oct 26;10(11):1692. doi: 10.3390/antiox10111692 (PMC8614663; doi:10.3390/antiox10111692)
Supplement: Supplementary file 1 [file antioxidants-10-01692-s001.zip › antioxidants-1429982-supplementary.pdf]

Next-generation ultrasol curcumin boosts muscle endurance and reduces muscle damage in treadmill-exhausted rats

Emre Sahin[1], Cemal Orhan[2], Fusun Erten[3], Besir Er[4], Manutosh Acharya[5], Abhijeet A. Morde[5], Muralidhara Padigaru[5], Kazim Sahin[2]*,

[1] Department of Animal Nutrition, Faculty of Veterinary Medicine, Bingol University, Bingol 12000, Turkey; esahin@bingol.edu.tr (E.S.)
[2] Department of Animal Nutrition, Faculty of Veterinary Medicine, Firat University, Elazig 23119, Turkey; corhan@firat.edu.tr (C.O.); nsahinkm@yahoo.com (K.S.)
[3] Department of Veterinary Science, Pertek Sakine Genc Vocational School, Munzur University, Tunceli, Turkey.; fusunerten@munzur.edu.tr (F.E.)
[4] Department of Biology, Faculty of Science, Firat University, Elazig 23119, Turkey; beshir.er@hotmail.com (B.E.)
[5] Research and Development, OmniActive Health Technologies, Mumbai, India; m.acharya@omniactives.com (M.A.); a.morde@omniactives.com (A.A.M); m.padigaru@omniactives.com (M.P.)

* Correspondence: nsahinkm@yahoo.com (K.S.); Tel.: +90-532-7473506 Phone: +904242370000/3938

A

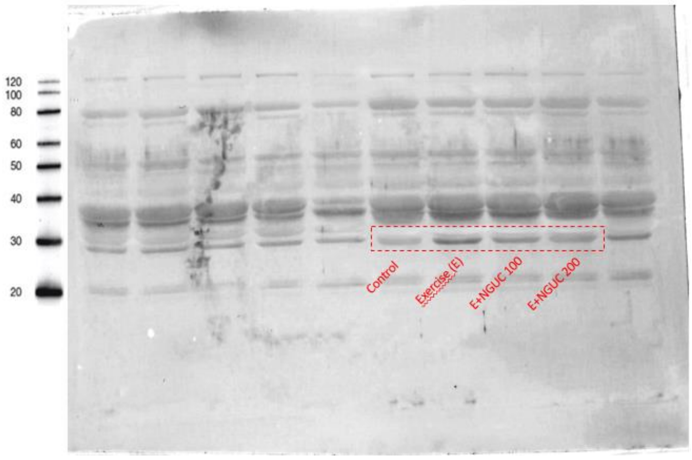
120
100
80
60
50
40
30
20
Control
Exercise (E)
E+NGUC 100
E+NGUC 200

B

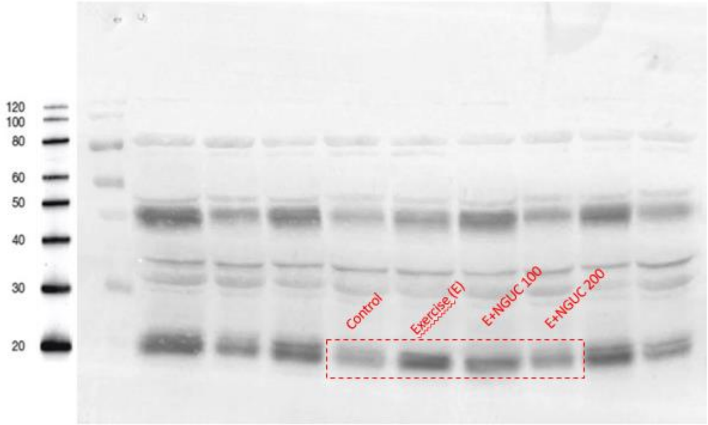
120
100
80
60
50
40
30
20
Control
Exercise (E)
E+NGUC 100
E+NGUC 200

C

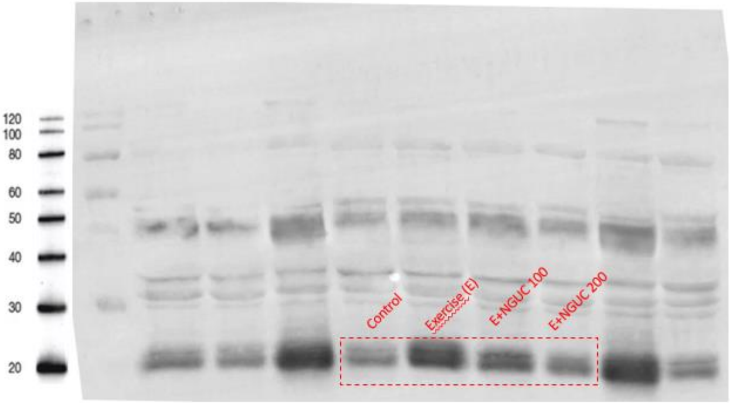
120
100
80
60
50
40
30
20
Control
Exercise (E)
E+NGUC 100
E+NGUC 200

D

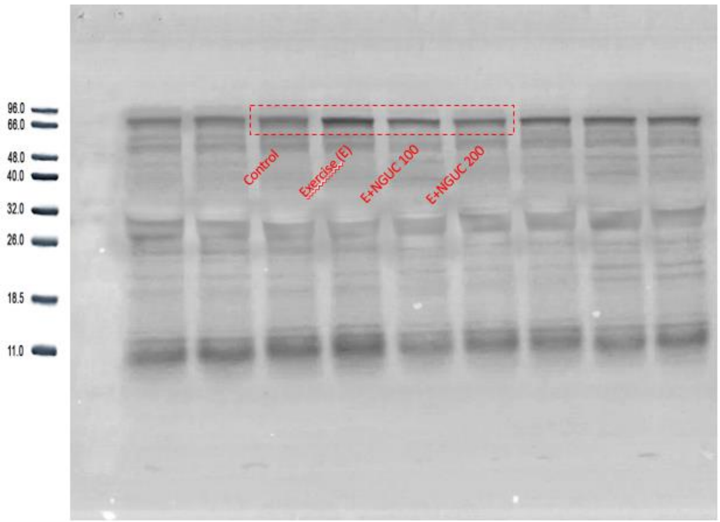

E

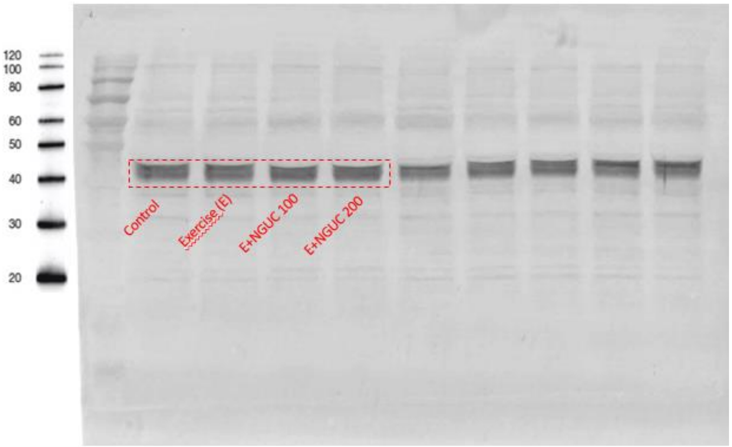

Supplementary Figure S1. Full immunoblots related to Figure 5 muscle tissue ((A) IL-1β,(B) IL-6, (C) TNF-α,, (D) PGC-1α, and (E) β-Actin). Each immunoblot is a representative of three independent experiments. Results shown in Fig.5 are delineated by red dotted rectangles. MW (in kDa) are indicated.

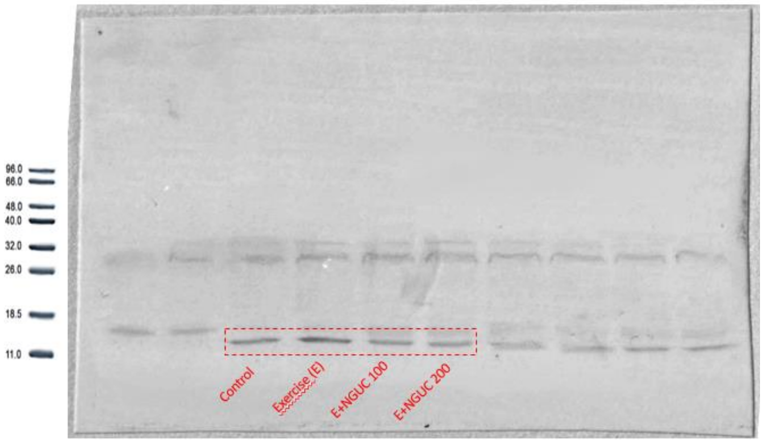

B

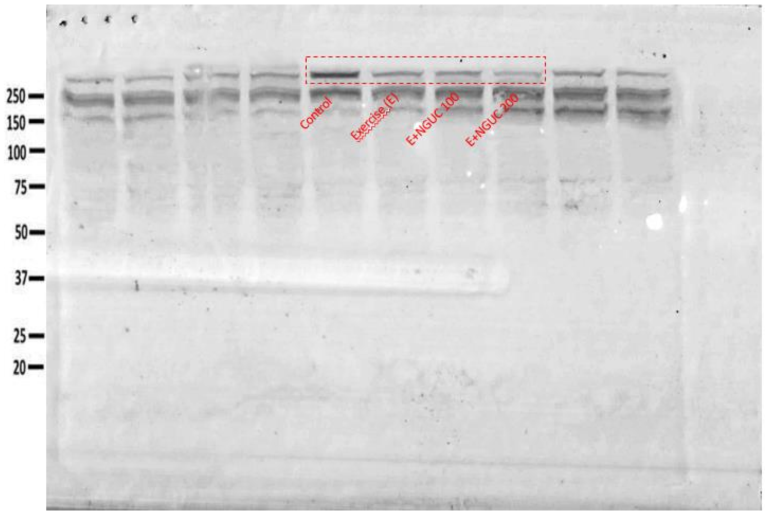
250
150
100
75
50
37
25
20
Control
Exercise (E)
E+NGUC 100
E+NGUC 200

C

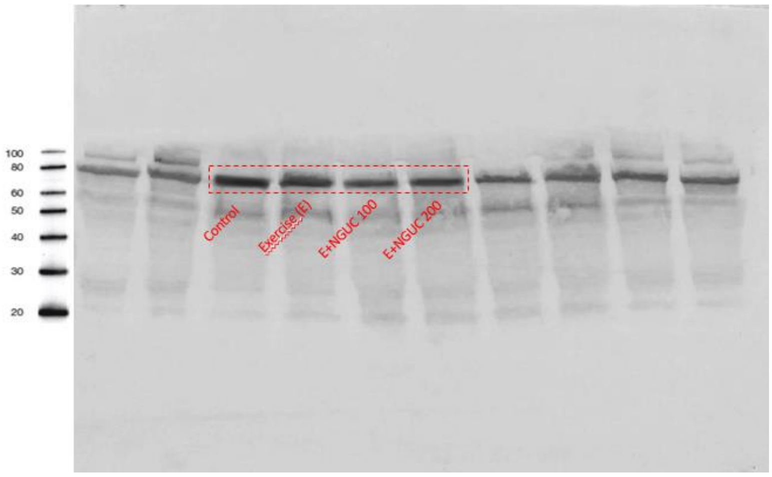
100
80
60
50
40
30
20
Control
Exercise (E)
E+NGUC 100
E+NGUC 200

D

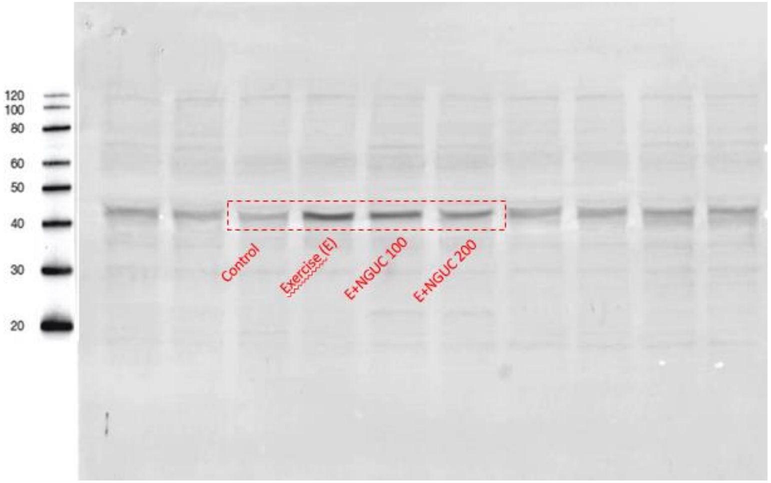
120
100
80
60
50
40
30
20
Control
Exercise (E)
E+NGUC 100
E+NGUC 200

E

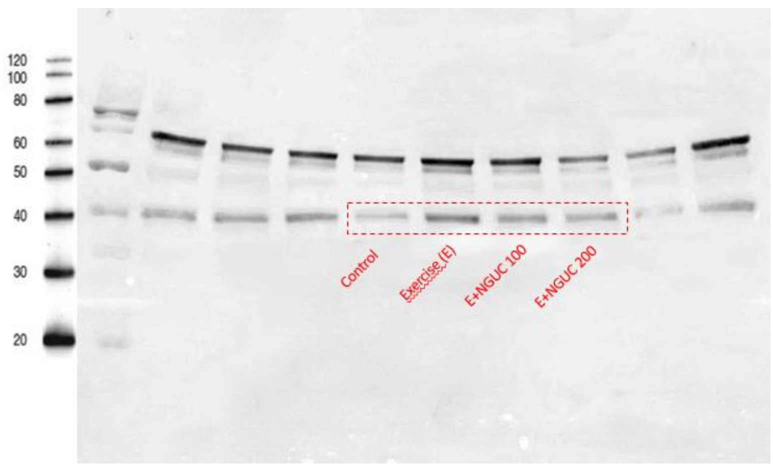

F

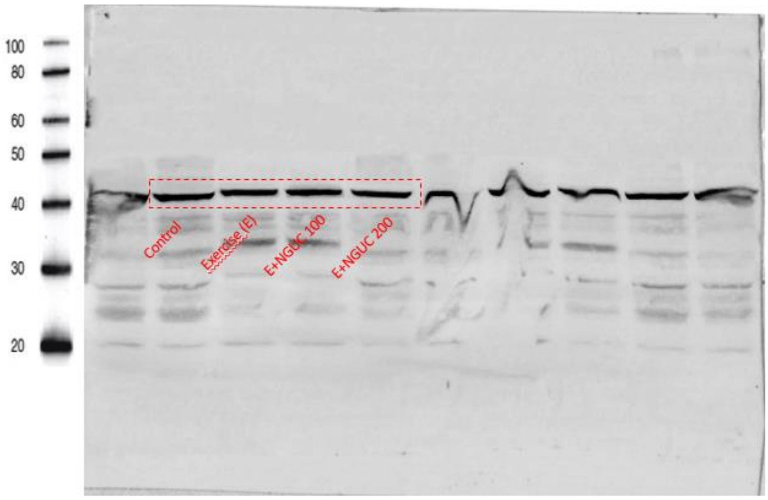

Supplementary Figure S2. Full immunoblots related to Figure 6 muscle tissue ((A) p-4E-BP1, (B) p-mTOR, (C) p-70S6K1, (D) MAFbx, (E) MuRF-1 and (F) β-Actin). Each immunoblot is a representative of three independent experiments. Results shown in Fig.6 are delineated by red dotted rectangles. MW (in kDa) are indicated.
